# Epileptic Asthma

**Published:** 1888-06

**Authors:** F. A. Harrington

**Affiliations:** 342 Fourteenth Street, Buffalo, N. Y.


### Translations.

### EPILEPTIC ASTHMA.

By F A. HARRINGTON, A. M., M. D.

*Journal de Medicine de Paris*, February 19, 1888.

Taking etiology as the basis of classification, we recognize four varieties of asthma, viz., that co-existing with chronic bronchitis, that with emphysema, our uncomplicated asthma, and cardiac asthma so-called. The last evidently is an intense form of dyspnœa, subordinate to the cardiac affection; but the course of the disease and the clinical history in each of the first two forms of asthma, are conclusive evidence that there exists two diseases, not an essential part of the asthma, but merely associated with it and characterized by sudden asthmatic paroxyms.

During recent years, I have had opportunity to observe several cases that came under my notice; and, after careful thought, I have not hesitated to add to the four universally recognized forms a fifth variety of asthma, to be designated *epileptic asthma*. After a recital of the facts, and an account of the observations, I trust that I shall have proven that the name has been appropriately applied.

In 1881, I published in *Concours Médical*, No. 51, an account of a neurosis of the pneumogastric nerve, with paroxysms varying in intensity, recurring irregularly, and similar to asthmatic attacks. These exacerbations occurred indifferently by day or by night, and lasted nearly twenty-four hours; they were accompanied by chills, which were succeeded by a sweating stage, and occasionally by vomiting. Perfect health followed the attacks. The patient was four years old, and the affection bore a closer resemblance to bronchial asthma than to that of Kopp. I diagnosed an epileptic neurosis, and the mode of treatment, instituted in accordance with that belief, has justified the correctness of my diagnosis.

I have subsequently seen two cases of the same trouble in old men. Here is the clinical history of one: F. G., a farmer, sixty years of age. He has a vigorous constitution, and, until the last few years, has enjoyed immunity from disease. In the haying season of 1884, he had a severe attack of acute bronchitis, which was treated by a physician with the ordinary therapeutic remedies, *e. g.*, tar preparations, creasote, expectorants, counter-irritants, *et cet.* The disease baffled all attempts to check its progress, and, soon lapsing into a chronic state, displayed violent asthmatic paroxysms.

During the winters of 1885 and 1886, the patient suffered no particular inconvenience; but the attacks reappeared in the following spring, longer in duration and more painful. Their occurrence was irregular; always by night.

I visited the patient for the first time on February 20, 1887, at three P. M., and, notwithstanding the attack had notably diminished in intensity, it had by no means ended. The patient was sitting on a chair, with body strongly inclined forward, and respiration loud and wheezing. He complained of a pain in the right side, and the arm and leg on the right were colder than those on the left side. The skin looked pallid and death-like and cyanotic in places. The lips were blue; the whole surface of the body was bathed in cold perspiration. No cardiac murmur was audible, but the heart-sounds were so accelerated that the long pause was completely abolished. The pulse was frequent and feeble. Throughout the whole expanse of the chest, a stethoscopic examination revealed sibilant râles. Expectoration was glairy; at times, mucous. Percussion was alike on both sides. The tongue was clean; appetite good; habitual constipation. After a few months, the pain passed from the right to the left side of the body. It may be described as a sort of *aura* premonitory of the impending attack. The patient described it as follows: a painful sensation was experienced in the head, in the left frontal region; thence it extended towards the nipple on the same side. "It twisted the nipple," to use his expression. The pain soon reached the left side of the abdomen. It continued one-quarter to one-half of an hour, compelling him to flex the head upon the breast; after this, an exceedingly intense dyspnœa, that was almost unbearable, commenced. The patient was obliged to arise quickly and to run to an open window for fresh air. When the paroxysm terminated, expectoration became very profuse; sometimes there was a mucous discharge from the right nostril, or vomiting of mucus. Experience had taught him that a little brandy swallowed in coffee during the attack lessened its duration. In rare instances, the migraine, which was marked at the outset, lasts several hours after the attack. When the paroxysm is ended, perfect health returns without exception.

The usual therapeutic agents were ineffectual in this troublesome disease. Oxalic acid mitigated the attacks, but did not prevent their recurrence; morphine was of no more avail. The iodides, administered as a preventive, not only had no effect on the progress of the disease, but seemed actually to exercise an injurious action.

It was then owing to the complete intermittency and other characteristics of the symptoms, as well as to the irregularity of their appearance, that I conceived the idea of combating them with the anti-epileptic treatment. My treatment has been bromide of potassium, in doses of six grams daily, in conjunction with six milligrams of picrotoxine and an equal amount of arseniate of soda. The effect was marvelous, and I have met with astonishing and unfailing success. The paroxysms were almost entirely suppressed at one stroke, as only a single attack occurred at the end of ten days, and it was much milder and, as it were, rudimentary.

What is the nature of these attacks from which F. G. suffered? I shall unhesitatingly designate them as asthmatic paroxysms, but the entire accompanying phenomena differentiate them from the customary attacks of asthma. There is absolute intermittency, and during the interval the patient presents no symptoms of asthma. Such a termination seldom occurs in the more ordinary varieties of asthma, in which you will observe a continual dyspnœa, characterized by paroxysmal exacerbations. They recur, it is true, by night oftener than by day, but at very varying periods: at one time after the lapse of several days, at another time after a number of weeks. Beginning in the night, they prolong themselves the following day, or even longer; they are ushered in by a painful sensation in the head, comparable to an epileptic *aura*. Their termination is marked sometimes by a nasal discharge, sometimes by emesis. Finally, the preventive treatment in vogue for asthma has no efficacy in these paroxysms, while they yield most promptly to the simplest anti-epileptic remedies.

If the neurosis under discussion differs from common asthma in many essential regards, it differs also from *angina pectoris* by a line of demarcation not less evident. In the latter, it is not merely pain that is experienced, but a feeling of impending syncope. Respiration is much less embarrassed; it is but slightly accelerated; nor is it accompanied by any wheezing or other abnormal sound, not even by the sibilant râle of bronchitis, nor of pulmonary infarction. The suffering is more marked by day, contrary to what is seen in asthma and in the case cited of F. G. Doubtless, there were in that case pains that had a similarity to those in *angina pectoris*, but their location, course, duration, and characteristics were essentially different; for they began in the face and ended in the abdomen, without localizing themselves in the præcordial space nor radiating into the left arm.

Moreover, they always preceded the paroxysms and, in some instances, continued after them; but they diminished in intensity, or even disappeared, during an attack, while the pain of *angina pectoris* is particularly sharp at the time of the paroxysm, so that the patient aptly compares it to pincers tearing the flesh. As to the duration of the attack, it is longer, by far, than that of *angina pectoris.*

If the respiratory phenomena were incomparably more marked than those of *angina pectoris*, the cardiac symptoms, on the other hand, were less noticeable or entirely absent. What, in reality, is the state of affairs in *angina pectoris?* At the outset of the attack, always sudden, circulation at first seems disturbed; for an instant, the heart ceases beating; then begin strong and rapid pulsations, which continue several minutes, and which an intense præcordial pain succeeds. Concurrently you notice, to begin with, an irregular, slow, and full pulse; soon it becomes accelerated and small. The beat is indistinct, rapid and fluttering. In short, the signs of arterio-sclerosis, so often a feature of *angina pectoris*, are entirely lacking in the case of F. G.; and, while death is the rule in this painful trouble, epileptic asthma has invariably yielded to appropriate treatment.

I have not, then, a case of every-day asthma, nor of *angina pectoris*. Had I made only a stethoscopic examination, I might have diagnosed a very acute capillary bronchitis; but, after studying the course of the malady, I immediately discarded that hypothesis as inadmissible. In fine, the sudden invasion, without any assignable cause; the duration much briefer than that of the accompanying bronchitis; the sudden disappearance at the military hour, so to speak; the inevitable and periodic return of symptoms, reëstablishment of customary health during the intervals, success foreseen and assured by anti-nervous medication—here, surely, is a grouping of signs belonging to a neurosis, I can confidently assert to an epileptic neurosis seated in the ultimate filaments of the pneumogastric nerve.

To sum up all, I will state that, in my opinion, there are true epilepsies that do not manifest themselves by the customary train of cerebral symptoms, as seen in a severe epileptic attack; but by certain symptoms of asthma, modified and aggravated by those of the epileptic neurosis, and relieved by the curative treatment of epilepsy, though unaffected by that of true asthma.

Physicians, up to the present time, have failed to differentiate between the epileptic neurosis and ordinary asthma.

It is conceded that we admit that nervous disturbances arise in asthma, particularly convulsive movements of divers parts of the body. Laënnee mentions the loss of consciousness. We are justified, it seems to me, after a consideration of the complex symptoms, in placing the disease in the group of epileptic neuroses.

The symptoms noticed in the patient to whom I have referred, prove that the paroxysms originated in the terminal filaments of the pneumogastric nerve in the lungs and stomach, and that the laryngeal nerves were exempt from any functional disturbance.

Precisely an opposite state of affairs exists, as we have determined, in an affection which we can compare with the preceding, as regards its nature and treatment efficacious to overcome it. I refer to *thymic asthma*, or the *asthma of Kopp*. In this, instead of the catarrhal symptoms of suffocation, spasm of the glottis takes place.

"Thymic asthma occurs in infancy, from the age of three weeks up to eighteen months. It is attended by bronchial spasms, which are paroxysmal; respiration suddenly stops, and you will perceive only a very short, high-pitched, and whistling inspiration; the air passes difficultly through the contracted glottis. The sharp cry, accompanying inspiration, is heard either at the commencement of the paroxysm, when it is soon stifled by suspension of respiration, or at the end, when the infant begins to breathe again. The cry is constant and pathognomonic of the disease. The other symptoms happening during the paroxysm result, naturally, from failure of respiration. The child flexes the trunk forcibly back, or, if the attack is severe, falls backward. The face has an expression of anxiety; it is livid at first, then pallid; nostrils dilated; eyes fixed; hands cold; thumbs pressed into the palms; evacuations may be involuntary. The attack lasts one-half, one, and sometimes two or three minutes; then the patient cries for a time and is uneasy, but soon regains his customary good spirits. During the intervals, the child is healthy, as much so as other children. The attack of suffocation begins, as a rule, when the infant awakes, cries, frets, or attempts to swallow quickly. At first the attacks take place at intervals of eight days or more, but, gradually becoming more frequent, they appear as often as ten to twenty times daily. Death commonly supervenes at this period in the middle of an attack which comes upon the infant when laughing and playing; but more often the patient passes to the second stage, that of *general epileptiform convulsions.* The epileptic attack and the asthmatic do not always coincide, but oftener alternate.'' (In *Medical Gazette*, 1836, p. 21.) We may, with propriety, add that the first paroxysm occurs indifferently by night or day, and that the disease is essentially chronic.

Dr. Kopp, of Copenhagen, and, more recently, Dr. Hirsch, of Königsberg, have ascribed it to hypertrophy of the thymus gland. Caspari and Pagenstecher, on the contrary, consider the alteration of volume, if existing, as an effect, and not a cause, of this infantile asthma, which they properly compare to an affection of the pneumogastric nerve.

The researches of Herard demonstrate that the thymus gland is sometimes small, sometimes enlarged, depending upon whether the infant is delicate or robust; but the degree of its hypertrophy makes no material difference as to the recurrence of the spasm of the glottis.

What, then, is the so-called thymic asthma?

Believing that the symptoms of that disease pointed clearly to an epileptic neurosis of the laryngeal filaments of the vagus nerve, I have observed, as a confirmation of that belief, in purely asthmatic attacks accompanied exclusively by spasm of the glottis, something analagous to epileptic vertigo, which precedes the convulsion, and I have marked another symptom of true cerebral epilepsy when, in the course of the attack, some general epileptiform spasms were added. Induced by these theoretical ideas, I became convinced that the treatment would be a good test; and only last year I had an opportunity to try the anti-epileptic treatment. The patient was an infant, ten months old, presenting symptoms of the asthma of Kopp. The result has justified my conceptions, for I have had the satisfaction of thoroughly uprooting the morbid process.

The prognosis of that disease, up to this time, was regarded as very unfavorable and invariably fatal whenever there were added any epileptiform convulsions. I am inclined to think, thanks to the new treatment, that, in general, we may entertain hopes of saving these little patients.

*Conclusions.*—A form of bronchial asthma is met with, that I consider merits the name of an epileptic neurosis of the gastro-pulmonary filaments of the vagus nerve.

That disease, up to the present time, has not been differentiated from ordinary asthma; it is characterized by varying paroxysms, succeeded by health. These attacks are preceded, in many instances, by pain in the head, comparable to an epileptic *aura*, and they happen by day, or last during it, vomiting being a frequent symptom. What particularly signalizes the affection, is that it is not improved by the routine treatment of asthma, but, on the contrary, successfully combated by that of epilepsy.

You will observe that the distinguishing characteristic of the new variety of asthma, that I am seeking to introduce into the nosological catalogue, has an important practical bearing; for it guides us to follow a new line of treatment, of remarkable efficacy.

The asthma of Kopp, in accordance with my hypothesis, can be nothing else than an epilepsy analogous to asthma, and I designate it as epileptic asthma. Its treatment is the same as that of epilepsy, as one observation has proven. It has this difference, that, instead of emanating from the gastro-pulmonary filaments of the vagus nerve, it has its seat of origin in the laryngeal filaments of the eighth pair of nerves. Such difference is of no practical importance.

342 Fourteenth Street, Buffalo, N. Y.

